# *BreakAlign*: a Perl program to align chimaeric (split) genomic NGS reads and allow visual confirmation of novel retroviral integrations

**DOI:** 10.1186/s12859-022-04621-1

**Published:** 2022-04-15

**Authors:** Emanuele Marchi, Mathew Jones, Paul Klenerman, John Frater, Gkikas Magiorkinis, Robert Belshaw

**Affiliations:** 1 Nuffield Department of Medicine, University of Oxford, Oxford, UK; 2 Department of Hygiene, Epidemiology and Medical Statistics, Medical School, National and Kapodistrian University of Athens, Athens, Greece; 3 Department of Biology, College of Science and Technology, Wenzhou-Kean University, Wenzhou, Zhejiang Province China

**Keywords:** NGS, Retrovirus, Provirus, Integration, Insertion, Detection

## Abstract

**Background:**

Retroviruses replicate by integrating a DNA copy into a host chromosome. Detecting novel retroviral integrations (ones not in the reference genome sequence of the host) from genomic NGS data is bioinformatically challenging and frequently produces many false positives. One common method of confirmation is visual inspection of an alignment of the chimaeric (split) reads that span a putative novel retroviral integration site. We perceived the need for a program that would facilitate this by producing a multiple alignment containing both the viral and host regions that flank an integration.

**Results:**

*BreakAlign* is a Perl program that uses *blastn* to produce such a multiple alignment. In addition to the NGS dataset and a reference viral sequence, the program requires either (a) the ~ 500nt host genome sequence that spans the putative integration or (b) coordinates of this putative integration in an installed copy of the reference human genome (multiple integrations can be processed automatically). *BreakAlign* is freely available from https://github.com/marchiem/breakalign and is accompanied by example files allowing a test run.

**Conclusion:**

*BreakAlign* will confirm and facilitate characterisation of both (a) germline integrations of endogenous retroviruses and (b) somatic integrations of exogenous retroviruses such as HIV and HTLV. Although developed for use with genomic short-read NGS (second generation) data and retroviruses, it should also be useful for long-read (third generation) data and any mobile element with at least one conserved flanking region.

**Supplementary Information:**

The online version contains supplementary material available at 10.1186/s12859-022-04621-1.

## Background

Retroviruses replicate by integration of their genome into a host chromosome. Over millions of years, integration into germline cells has led to endogenous retroviruses (ERVs) making up 8% of our—and other mammal—genome sequences [1], and exogenous retroviruses (XRVs) such as HIV and HTLV can persist in replicating somatic cells. Detecting the integration sites of XRVs and non-reference (not in the host reference genome) ERVs, and other transposable elements is hindered by (a) the sequence similarity of any one integrated element to many others and (b) approximately half the genome is composed of transposable elements so the flanking regions of many integrations are similar to other genomic regions. These problems have resulted in 20 programs designed to detect and report the genomic coordinates of integrations [2]. A benchmarking study using non-reference transposable elements structurally similar to retroviruses [3] showed marked differences in performance with common problems in precision (false positives). Vy-PER [4] and STEAK [5] are additional recent additions specifically for retroviral integrations. The gold-standard validation of non-reference integrations involves cloning [6] but researchers often use visual inspection and present graphical images of the read alignment with the viral LTR (Long Terminal Repeat) and TSD (Target Site Duplication) [7–10]. The presence of a TSD is strong evidence of an integration. We perceived the need for a program to facilitate this visual validation and present *BreakAlign* as a stand-alone program, having written a prototype as the final confirmatory step in a pipeline to detect non-reference integrations in one lineage of ERVs—Human Endogenous Retrovirus type K (HERV-K) [9]. We now also use *BreakAlign* to measure clonal expansion of somatic cells carrying HIV-1 integrations (unpublished data).

*BreakAlign* provides visual validation of novel retroviral integrations (i.e. ones not in the reference genome sequence), whose putative coordinates have been output from one of the many detection programs. The input is NGS (Next—or second—Generation Sequencing) reads from whole genome sequencing (WGS) with or without experimental [11] or bioinformatic (in silico) enrichment procedures [9]. Briefly, *BreakAlign* uses *blastn* to compare reads to the virus and host genome and extract those chimaeric ('split') reads that contain both viral and host DNA sequences (i.e. which span the input integration site). The program then presents these reads in a multiple alignment to the genomic region. (In addition to validating the integration, this file could for example also be used to measure clonal expansion.)

## Implementation

### Overview

**Fig. 1:**
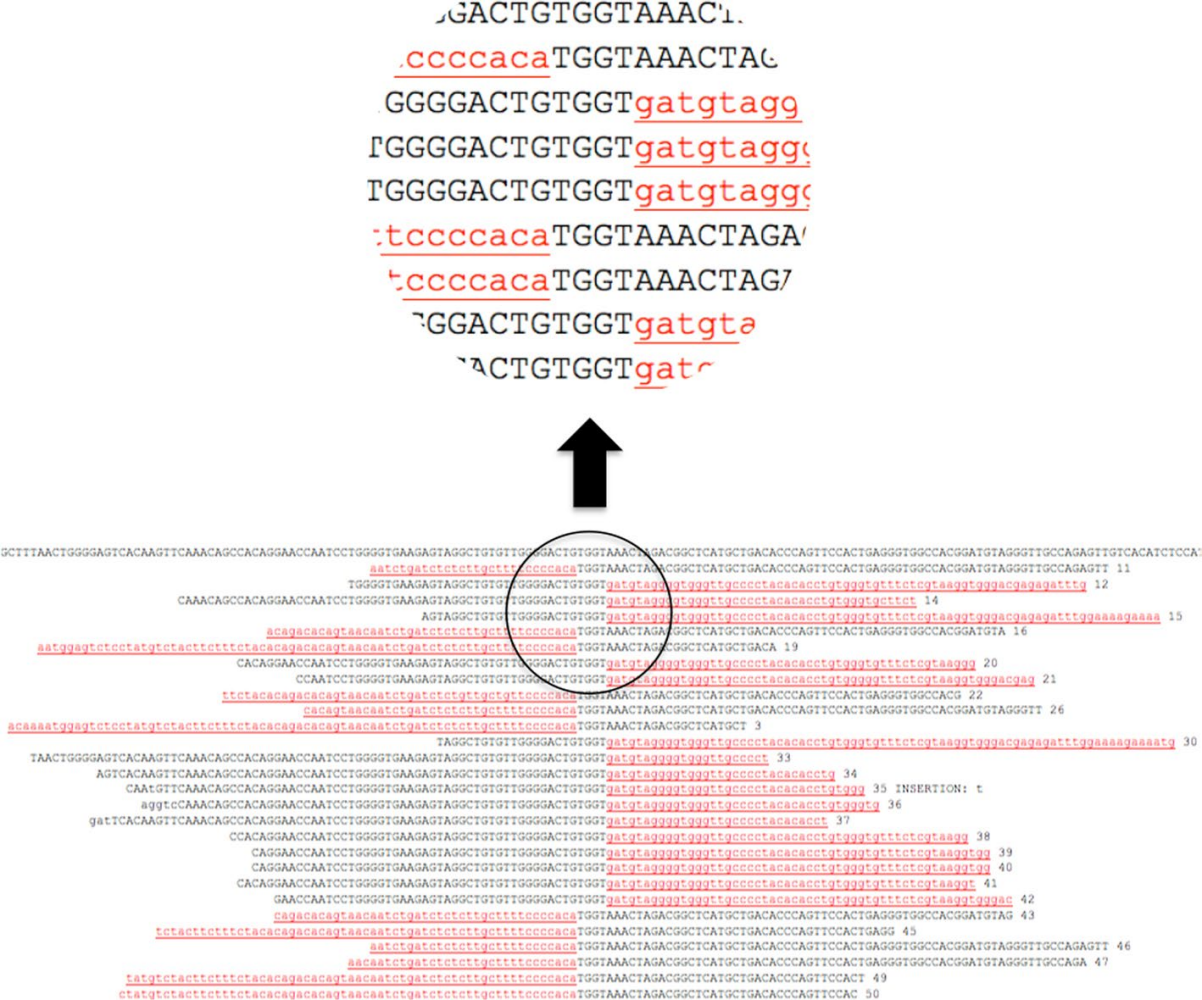
Sample output from *BreakAlign* showing an illustrative HERV-K integration. The top line is the section of the host genome spanning the integration site. Numbered sequences below show individual NGS reads aligned to the host genome with viral DNA in lower case, red and underlined (viral sequences on the left are the 3′ end of the 3′ LTR; viral sequences on the right are the 5′ end of the 5′ LTR). Reads containing insertion/deletions (indels) are indicated, with insertions and substitutions shown in lower case and deletions shown as hyphens. The intervening 'TGGT' motif is the TSD—see Marchi et al. [9] for a detailed explanation of how the TSD is formed and the adjacent HERV-K LTR sequences in both forward and reverse orientation integrations. Note, in this integration there is a known A to G substitution in the upstream TSD [7, 8] which leads to the characteristic six nucleotide HERV-K TSD—in this case TGGTAA—appearing to be shortened to only four nucleotides. Image shown is the output HTML file, and a text version without colours or underlining is also output

*BreakAlign* is a Perl program intended to be run in UNIX (including Mac OSX—not Mac Desktop Cloud) that uses *blastn* to find chimaeric sequences within a FASTA NGS read file and align them to the host genome sequence (Fig. 1). In this alignment, the existence of a TSD (Target Site Duplication) and identity of host flanking and viral sequences confirms a true integration rather than a sequencing artefact or alignment error. *BreakAlign* is downloadable from https://github.com/marchiem/breakalign together with the files necessary for a test run using the command line example below. Program options are summarised below (also in Additional file 1; 'Program Options') and can be shown with the -help switch.

### Sample command line

$ perl breakalign.pl -f chr19_29855576_29856018.fa -r NGSreads.fa -vr LTR.fa

The above sample LTR, genome sequence and read files are with breakalign.pl on the GitHub site and will generate the alignment in Fig. 1. For a test run using HIV-1 instead of HERV-K, use the following command line (Additional file 1: Fig. S1; data from [11]. The read file (NGSreads.fa) is a toy dataset: any real FASTA-format NGS file can be used here.

$ perl breakalign.pl -f chr21_14511311_14511999.fa -r NGSreads.fa -vr LTR-HIV.fa

### Executing the program

#### Program details

**Fig. 2:**
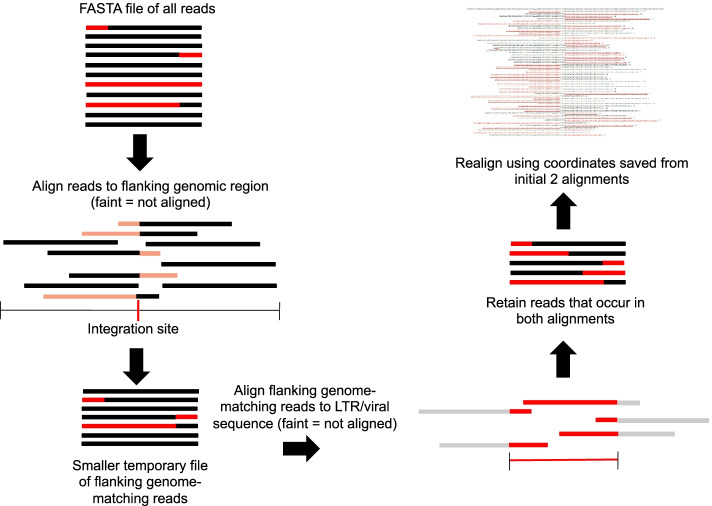
Flow chart illustrating the basic steps within *BreakAlign*. Parts of chimaeric reads that align to the virus are shown in red. Generating the local *blastn* database can be time-consuming (see text). Other stages in *Breakalign* will typically take a minute or less

*BreakAlign* runs from the command line in UNIX operating systems (including Mac OSX) with Perl 5. It was designed on a Linux machine (Ubuntu 16.04) with *blastn* version 2.2.31+, perl subversion v5.22.1. We have also tested it on *blastn* version 2.2.27+. *BreakAlign* generates a local database (using *makeblastdb*) of the read file to make a computationally more efficient alignment of reads (used as subjects) to the host reference region (used as query sequence). The BioPerl module *SeqIO* is required only if the human reference genome is to be used (we developed *BreakAlign* using BioPerl core modules v1.6.924-3). The steps in *BreakAlign* are illustrated in Fig. 2. The program requires four inputs listed below.

1. *BreakAlign* runs *blastn* in NCBI's BLAST + package (the earlier *blastall* version will not work). *BreakAlign* will check for a system *blastn* installation or you can provide a directory path to a local *blastn* installation using either the -bp switch or writing the *blastn* directory into line 20 of the script (paths must end in /bin/).
2. A FASTA file of the viral LTR sequence indicated by the -vr switch. An LTR sequence for HERV-K is provided on the GitHub site.
3. An NGS read file in FASTA format indicated by the -r switch. *BreakAlign* can be used with WGS files but works best with pre-filtered read files from bioinformatic "enrichment"—filtering out reads that map well to the host reference. In Additional file 1 ('NGS read preparation’), we provide guidance on how to reduce the size of WGS BAM files—taking advantage of the fact that the desired chimaeric reads will not map well to the reference genome. Smaller read files can also be generated using an enriched sequencing protocol, *e.g.* where the library is generated from DNA hybridizing to viral LTR probes. Building the initial *blastn* database is the only time-consuming part. Help to convert these files to FASTA format, and to combine paired-end FASTQ files into a single FASTA file, is also given in Additional file 1.
4. The putative integration site can be input in one of two ways. (a) An approximately 500nt human genomic sequence in FASTA format that spans the putative integration site can be input using the -f switch (see sample command line). This length will allow all chimaeric NGS reads, both up- and downstream of the integration, to be arranged in a multiple alignment such as the one shown in Fig. 1. (b) *BreakAlign* can retrieve automatically the reference sequence spanning the putative integration site from an installed copy of the reference human genome. This genome must be downloaded from either the UCSC website (http://hgdownload.soe.ucsc.edu/goldenPath/hg19/chromosomes/) or from the NCBI–NIH genomes FTP site (ftp://ftp.ncbi.nih.gov/genomes). The download will contain chromosomes as individual FASTA file within in a single folder (chr1.fa, chr2.fa, etc.). To use this option the following are also required.
  The BioPerl module SeqIO must be installed (see Additional file 1, 'Program Requirements').
  Indicate the directory path where the host reference genome sequence is stored using the -fr switch (/path/to/reference_folder/).
  Either
    - a single coordinate range spanning the putative integration site can be provided as a string in the format ‘chrN:start–end’ using the -c switch (e.g. chr16:89,577,447–89,578,013). We suggest approximately 500nts.
    - or a bed file with multiple coordinates can be provided using the -bf switch. The following sample command line will recover both the HERV-K chromosome 19 integration illustrated in Fig. 1 plus another in chromosome 5 (Additional file 1: Fig. S2) using the toy chromosome sequences in the Human_ref folder in GitHib. By default *BreakAlign* will build a new *blastn* database for each coordinate, which can be time consuming, so the -kd switch will keep and reuse this database (you will need to delete this All_reads_db database manually at the end if you do not wish it to be reused in the next run).

$ perl breakalign.pl -bf coord.bed -r NGSreads.fa -vr LTR.fa -fr Human_ref/

#### Program options

A full list is provided in Additional file 1 ('Program Options') and via the -help switch. The key ones are as follows.

The following two are necessary for all runs (plus paths if the file is not in the same directory as *BreakAlign*).

-vr File with the viral (LTR) sequence.
-r The NGS read file.

One (only) of the following is required.

-f This is the file with the user-supplied reference sequence.
-fr Path to the reference folder containing a FASTA file for each chromosome.

One of the two following options is also required with the -fr switch.

-c Text giving a single genome coordinate in the form < Chr> : < Start>- <Stop> (e.g. chr16:89577447–89578013).
-bf A file in bed format containing multiple coordinates to test.

If multiple integrations are being tested using the same NGS read file, either using the -bf switch or entering coordinates manually, then the -kd switch will allow the program to reuse the initial *blastn* database (from *makeblastdb*) in the subsequent run(s). The most time-consuming part of running *BreakAlign* is building this initial database of all reads against the genome sequence flanking the putative integration.

## Results and discussion

In addition to validating the integration, the aligned reads from *BreakAlign* can be further processed and counted, e.g. in analyses of enriched NGS sequencing (following sonication) of blood cell DNA to measure clonal expansion in relation to development of disease in HTLV-1 infection [12] and persistence despite drug therapy in HIV-1 infection [13]. We have used *BreakAlign* in the manner described above to confirm long-term persistence of specific HIV-1 integrations in patients (data not shown).

This output can also be used for distinguishing between the homozygous and heterozygous status of ERV integrations by searching for non-chimaeric reads spanning the integration site, which will be either absent or of similar abundance [9].

The capture of chimaeric reads can be enriched through the employment of various laboratory protocols and/or by in silico enrichment from WGS libraries. Such target enrichment typically produces in the order of a million reads compared to > 100 million in a typical TCGA (NCI's The Cancer Genome Atlas) library, which are very time-consuming to process. As suggested in “Program details” above, removing all reads with a good match to the reference genome (which are therefore unlikely to be chimaeric) from whole genome BAM files will produce a much smaller FASTA read file (< 40 GB) that *BreakAlign* will process in under 2 h on most LINUX or Mac computers. Most of this time is used creating the initial *blastn* database, and using the -kd switch to retain this database will allow rapid validation of multiple putative integrations.

The advantages of *BreakAlign* over standard read-mapping and visualisation software are that (a) we can for the first time readily inspect the contiguous nucleotide sequences of the TSD and LTRs and (b) *BreakAlign* recovers more chimaeric reads, e.g. the default setting requires only 10 nucleotides to match the human reference genome and 10 to match the viral reference sequence. In Additional file 1 ('Comparison with existing software') we give examples of *BreakAlign* recovering over 50% more chimaeric reads than standard aligners*.*

*BreakAlign* has been written to confirm retroviral integrations, and its requirement of an initial 10nt exact match to the LTR will prevent it working on extremely old or fragmentary integrations. It should, however, work with any transposable element that has conserved flanking regions, which could be used instead of an LTR file. Similarly, retroviral integrations in non-human genomes could be analysed (if coordinates and a reference genome are used, these will need to use our chromosome-based nomenclature).

Finally, we look forward to development of accurate long-read (third generation) technology, which will make working with retroviral integrations much easier because such reads are capable of spanning the full integration. *BreakAlign* should work with long-reads and, as with the other possible uses mentioned above, we welcome future collaboration. We anticipate that the mapping and base quality of individual reads will be more of an issue with long reads (*BreakAlign* does not at present consider these). Lowering the length of the starting exact match required against the genome reference and LTR (-ws and -ws2 respectively) may be necessary. Debladis et al. [14] give an example of detecting novel integrations in *Arabidopsis* using MinIon reads, including what may be the first example of a complete LTR-retrotransposon (a selfish genetic element similar to an endogenous retrovirus) integration recovered in a single sequencing read.

## Conclusions

Confirmation of retroviral integrations that are not in the reference genome—both unfixed germline integrations of endogenous retroviruses or somatic integrations of exogenous retroviruses—require either individual PCR or observation of the resulting LTR ends with the characteristic TSD. We have created *BreakAlign* to allow the user to use the latter approach and confirm the presence of a novel retroviral integration in WGS NGS data.

## Availability and requirements

**Project name:** BreakAlign

**Project home page:** https://github.com/marchiem/breakalign

**Operating system(s):** UNIX

**Programming language:** Perl

**Other requirements:** BLAST+

**License:** GNU GPL

**Any restrictions to use by non-academics:** No.

## Supplementary Information

**Additional file 1.** Pdf format file with more detail on using *BreakAlign* plus **Figs. S1.** and **S2.**

## Data Availability

All data generated or analysed during this study are included in this published article's online additional files.

## Abbreviations

ERV: Endogenous Retrovirus
HERV-K: Human Endogenous Retrovirus type K (HML2)
HIV: Human Immunodeficiency Virus
HTLV: Human T cell Lymphotrophic Virus
LTR: Long Terminal Repeat
NGS: Next Generation Sequencing
TCGA: NCI's The Cancer Genome Atlas project
TSD: Target Site Duplication
WGS: Whole Genome Sequencing
XRV: Exogenous Retrovirus (free-living, horizontally transmitted)
