# Supplementary material for: BreakAlign: a Perl program to align chimaeric (split) genomic NGS reads and allow visual confirmation of novel retroviral integrations
Source: BMC Bioinformatics. 2022 Apr 15;23:134. doi: 10.1186/s12859-022-04621-1 (PMC9013057; doi:10.1186/s12859-022-04621-1)
Supplement: Supplementary file 1 — Additional file 1. Pdf format file with more detail on using BreakAlign plus Figs. S1. and S2. [file 12859_2022_4621_MOESM1_ESM.pdf]

Additional File 1

BreakAlign: a Perl program to align chimaeric (split) genomic NGS reads and allow visual confirmation of novel retroviral integrations

Emanuele Marchi*[,1], Mathew Jones[1], Paul Klenerman[1], John Frater[1], Gkikas Magiorkinis[2] and Robert Belshaw*[,3]

[1]Nuffield Department of Medicine, University of Oxford, Oxford, United Kingdom
[2]Department of Hygiene, Epidemiology and Medical Statistics, Medical School, National and Kapodistrian University of Athens, Athens, Greece
[3]Department of Biology, College of Science and Technology, Wenzhou-Kean University, Wenzhou, Zhejiang Province, China

*Correspondence: emanuele.marchi@ndm.ox.ac.uk, rbelshaw@kean.edu

Comparison with existing software

In figure S3 we show a non-reference integration site with all WGS (whole genome sequencing) reads mapped to the human reference genome using *NovoAlign* (http://novocraft.com/) and *IGV* (Thorvaldsdóttir *et al.*, 2013). The region shown contains the same integration site used in the tutorial and illustrated in figure 1 of the main text. In the centre of the image, the plot of read coverage immediately above the individual reads shows the characteristic increase over the 4-6 nucleotide region of the TSD, a region which is represented approximately twice as often as the surrounding genome because it is in both up- and downstream orientated reads. The chimaeric reads that span the integration site are only partially aligned by *NovoAlign* because the viral parts of these reads (which – of course – do not match the reference genome here) are not being shown by the aligner. Although such an alignment is suggestive of the integration it is not definitive. Also, in a heterozygous ERV locus (the one shown is homozygous) we would also have reads spanning the integration site making the integration even harder to discern. The advantages of *BreakAlign* over *NovoAlign* plus *IGV* are that (a) we can readily inspect the contiguous nucleotide sequences of the TSD and LTRs, (b) *BreakAlign* does not require removal of the viral part of chimaeric reads in order to optimally align them to

the reference genome, and (c) *BreakAlign* recovers more chimaeric reads (with as few as 10 nucleotides matching the reference genome – our default setting). In the example shown in figure S3, *NovoAlign* recovered only 28 reads that spanned the integration site compared with the 52 reads recovered by *BreakAlign* (Fig. 1).

A second example is using a HIV integration on chromosome 7, derived from the experimental enrichment of such reads in Sunshine *et al.* (2017). Reads were filtered using the same pipeline in that study, namely retaining reads that align using BWA (BWA-MEM) to both to the human and HIV genomes. This recovered 93 reads while *BreakAlign* retrieved 139 chimeric reads from their raw dataset (Fig. S4).

NGS read preparation

The input for *BreakAlign* is a single file of candidate NGS reads that need to be evaluated as authentic chimaeric reads produced by retroviral insertions. This file needs to be in FASTA format (because required by *makeblastdb*). In order to speed up the analysis of large WGS file (> 100MB) considerably, we routinely exclude all reads from the BAM file with CIGAR values equal to or above 95% of the read length, e.g. excluding reads with CIGAR values of 95 or more from 100bp-long read files. This will have the effect of removing all reads that match the reference genome well and hence will not contain chimaeric virus/host sequences from the flanks of the novel integration. Typically, this will reduce a WGS file to under 40GB FASTA input file (~10^8 100bp reads), which would take around 2 hours to build the BLAST database on a standard LINUX computer. For example, one WGS BAM file in our earlier Marchi *et al.* (2014) paper was reduced to 25% of its size: from 98GB to 25GB, with no loss of chimaeric reads. A ~5GB file would take only 7 minutes to process. Our procedure uses *samtools, Perl*, bedtools (*bamtofastq*) and the Unix shell programs *sed* ('stream editor') and *cat* ('concatenate') in the following three commands.

```
$ samtools view -h input_name.bam | perl -lane '$l = 0; $F[5]=~
s/(\d+)[MX=DN]/$l+=$1/eg; print if $l<95 or /^@/' | samtools view -bS - >
output_name.bam

$ bamtofastq -i output_name.bam -fq  output_name.fastq

$ sed -n '1~4s/^@/>/p;2~4p'   output_name.fastq >   output_name.fasta &
```

FASTQ format can also be converted into FASTA format using many freely available tools (e.g. FASTQ-to-FASTA converter in the Galaxy project, https://usegalaxy.org). It can also be done using the following shell command, which passes the output of a

cat command concatenating two paired-end FASTQ files to a *sed* command for conversion into FASTA format.

```
$ cat R1.fastq R2.fastq | sed —n '1~4s/^@/>/p;2~4p' — > your_reads.fasta
```

Program Requirements

BreakAlign runs from the command line in UNIX operating systems (including Mac OSX) with Perl 5 and *BLAST+.* The required *blastn* and *makeblastdb* programs are available from https://www.ncbi.nlm.nih.gov/books/NBK279671 (note, *blastn* will cope with reverse-complemented sequences). The BioPerl module *SeqIO* is also required if the human reference genome is to be used. This module can be installed on most systems by the following two commands.

```
$ perl -MCPAN -e shell
$ install Bio::SeqIO
```

Program options

Running *BreakAlign* solely with the switch -help with bring up the following options.

-f FILE. This is the user supplied reference sequence.
-fr FOLDER containing FASTA files for each chromosome.
-vr FILE. This is the LTR reference sequence.
-c STR. Genomic coordinates in the form <Chr>:<Start>-<Stop> (e.g. chr16:89577447-89578013).
-r FILE containing sequences reads.
-ws INT. Minimum word size alignment of reads to the reference sequence (default is 10).
-ws2 INT. Minimum word size alignment of reads to the LTR sequence (default is 10).
-bp PATH to blastn installation.
-kt Keep all the temporary files created for a single integration site inspection.
-bf FILE in bed format containing multiple coordinates to test.
-kd Keep the BLAST reads database files (in case of a very large input read file).
-nt INT. Number of threads to use in the blastn query command.

Sensitivity. The minimum word size for the *blastn* aligning can be specified using the —ws and —ws2 switches for host and LTR sequences respectively. The default is 10 for —ws2 and 10 for —ws2. Using a smaller word size can sometimes allow detection and alignment of additional chimaeric reads.

Supplementary Reference

Thorvaldsdóttir, H., Robinson, J.T. and Mesirov, J.P. 2013. Integrative Genomics Viewer (IGV): high-performance genomics data visualization and exploration, *Brief. Bioinform.*, 14: 178-192.

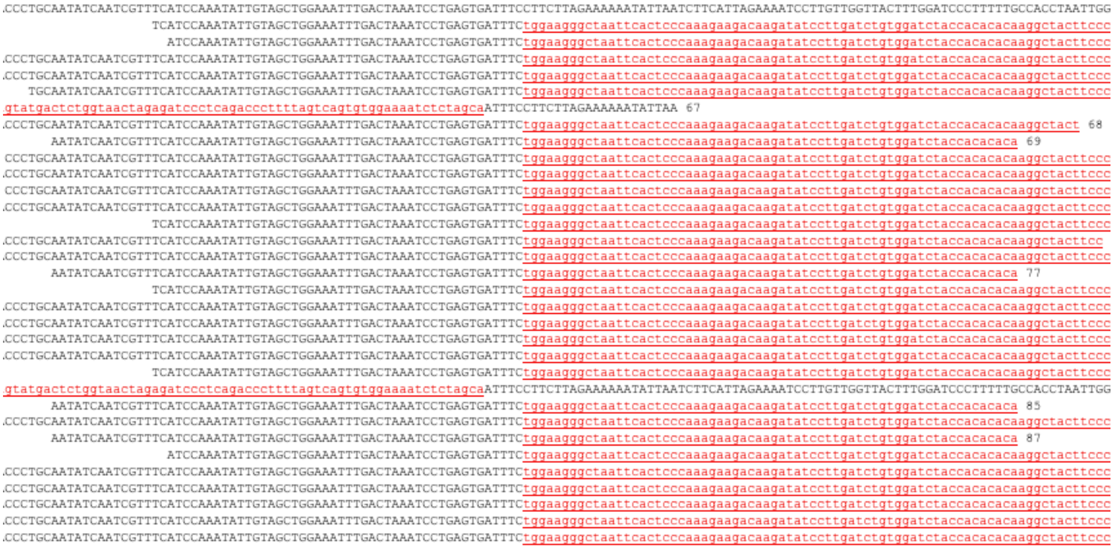

Figure S1. Sample output from *BreakAlign* showing an illustrative HIV-1 integration. See main text figure 1 legend for explanation.

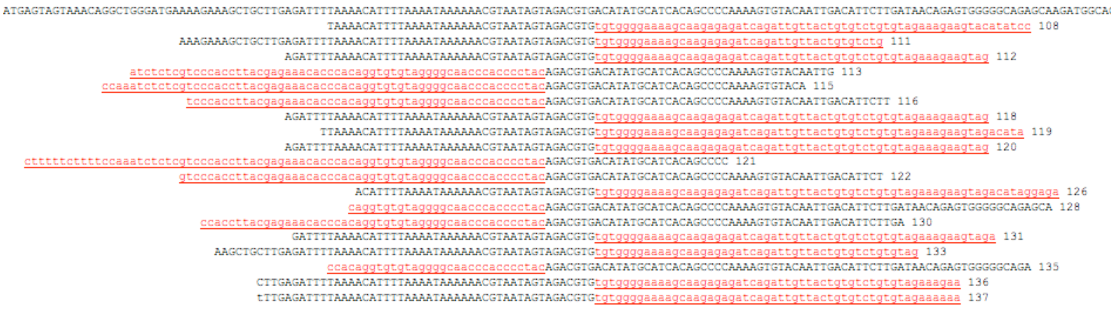

Figure S2. Second integration in the toy dataset used in the main text to illustrate the processing of multiple integrations using coordinates in a bed file.

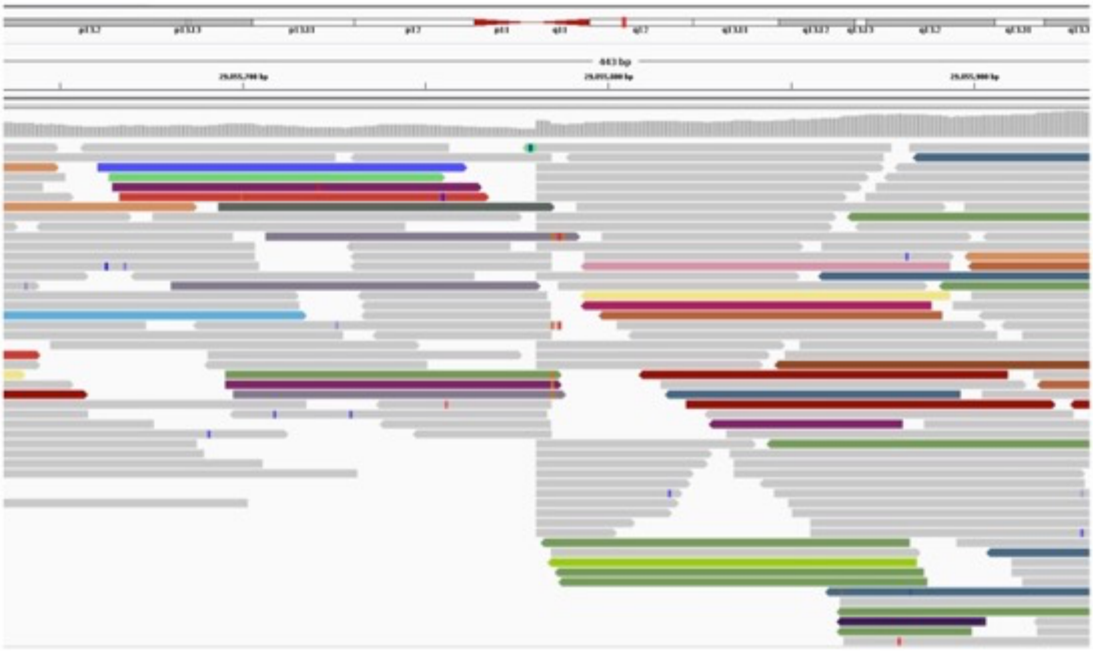

Figure S3. Region of integration site used in figure 1 with full WGS file mapped to hg19 using *NovoAlign*. Note characteristic increased coverage of the 4nt TSD in the centre. Image is a screenshot is from *IGV*.

A)

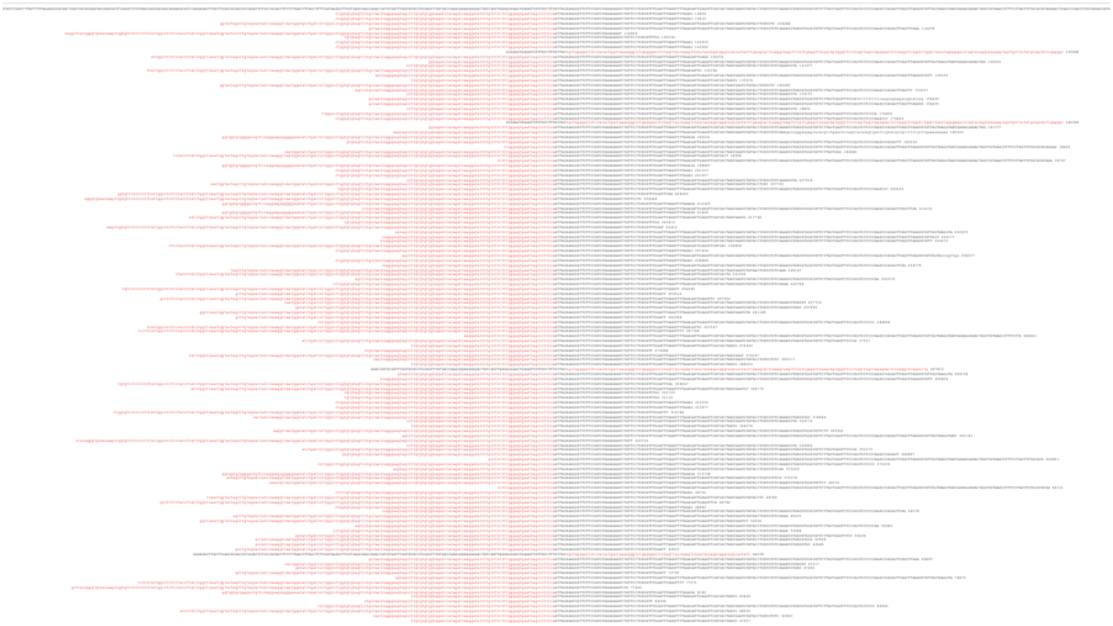

B)

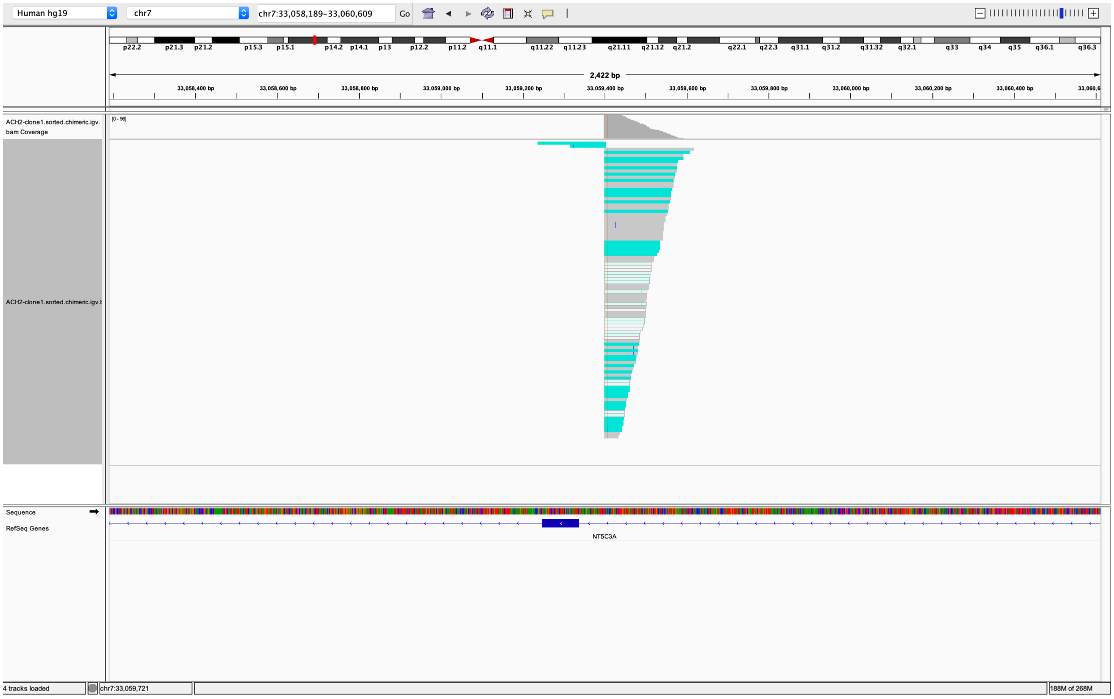

Figure S4. A) BreakAlign output with 139 chimaeric reads. B) 93 chimaeric reads found by BWA and displayed in IGV.
